# Place of death of children and young adults with a life-limiting condition in England: a retrospective cohort study

**DOI:** 10.1136/archdischild-2020-319700

**Published:** 2020-12-21

**Authors:** Deborah Gibson-Smith, Stuart William Jarvis, Lorna K Fraser

**Affiliations:** Health Sciences, University of York, York, UK

**Keywords:** palliative care, epidemiology, mortality

## Abstract

**Objective:**

To assess trends in place of death for children with a life-limiting condition and the factors associated with death at home or hospice rather than hospital.

**Design:**

Observational cohort study using linked routinely collected data.

**Setting:**

England.

**Patients:**

Children aged 0–25 years who died between 2003 and 2017.

**Main outcome measures:**

Place of death: hospital, hospice, home. Multivariable multinomial logistic regression models.

**Results:**

39 349 children died: 73% occurred in hospital, 6% in hospice and 16% at home. In the multivariable models compared with dying in a hospital: neonates were less likely, and those aged 1–10 years more likely, than those aged 28 days to <1 year to die in hospice. Children from all ethnic minority groups were significantly less likely to die in hospice, as were those in the most deprived group (RR 0.8, 95% CI 0.7 to 0.9). Those who died from 2008 were more likely than those who died earlier to die in a hospice.

Children with cancer (RR 4.4, 95% CI 3.8 to 5.1), neurological (RR 2.0, 95% CI 1.7 to 2.3) or metabolic (RR 3.7, 95% CI 3.0 to 4.6) diagnoses were more likely than those with a congenital diagnosis to die in a hospice.

Similar patterns were seen for clinical/demographic factors associated with home versus hospital deaths.

**Conclusions:**

Most children with a life-limiting condition continue to die in the hospital setting. Further research on preferences for place of death is needed especially in children with conditions other than cancer. Paediatric palliative care services should be funded adequately to enable equal access across all settings, diagnostic groups and geographical regions.

**What is already known on this topic?**

- Place of death is used as a quality measure of end-of-life care.
- More children die at home or in hospice setting if they have palliative care input.
- The current evidence states that most parents, children and providers prefer home death.

**What this study adds?**

- The vast majority of children with a life-limiting condition continue to die in the hospital setting.
- Children with a cancer diagnosis are much more likely to die at home or in hospice than children with other life-limiting conditions.

## Introduction

Although deaths in childhood have been decreasing, there are still 4500 infants and children who die in England and Wales every year^1^ and approximately 50% of deaths in children^2^ are for children with a life-limiting or life-threatening condition. Access to palliative and end-of-life care are therefore important components of paediatric health services.^3^

Palliative care services for children and young people in the UK have developed locally with heavy reliance on individual clinicians and third sector organisations such as children’s hospices.^3^ That ad hoc provision means delivery of palliative care is often ‘inconsistent and incoherent’.^4^ The recent NICE guidelines^5^ and quality standards of providing care^6^ include: ‘Infants, children and young people approaching the end of life and being cared for at home have 24 hour access to both children’s nursing care and advice from a consultant in paediatric palliative care’. Currently in England, while there are more than 50 children’s hospices, there are even tertiary children’s hospitals, including oncology centres, without a paediatric palliative care service.

Place of death has been used in policy documentation as a measure of quality of palliative or end-of-life care in developed countries such as the UK.^7^ The assumption that everyone wants to die at home has been contested in both children’s and adult palliative care in recent years.^8 9^ A recent review has concluded that ‘Most parents, children, and providers prefer home death and the long-term outcomes for parents (even 6–8 years after the death of their child) may be better when their child died at home’ but the authors also acknowledge that not all parents prefer a home death and the evidence is often from small studies.^10^ However, at a population level, if we are truly able to offer choice of place of care at the end of life then we should see a spread between the possible places of death. One of the aims of recent NHS England service specification for palliative care is that ‘more children and young people will achieve their preferred place of care at the end of their lives’. A national study showed that children who had palliative care input were eight times as likely as those without palliative care input to die in the community rather than in hospital.^11^ Therefore, the distribution of deaths between hospital, home and hospice may provide an indication of the degree to which families are able to access palliative care.

### Aims and objectives

This study aims to assess the trends in place of death for children who died with a life-limiting condition in England from 2003 to 2017 and the clinical and demographic factors which are associated with death at home or hospice rather than hospital.

## Methods

### Data sources

Linked individual-level inpatient Hospital Episode Statistics (HES) and Office for National Statistics (ONS) death records were obtained from NHS Digital. These two data sets were linked by NHS Digital based on National Health Service number, gender date of birth and postcode.^12 13^ Children and young people with a life-limiting condition were identified by matching recorded diagnostic codes in inpatient records against the previously developed ICD-10 coding framework^14^ (online supplemental table 1), for individuals aged 0–25 years (1 January 2000 to 31 December 2017).

10.1136/archdischild-2020-319700.supp1 Supplementary data

### Data management

Place of death was categorised as hospital, hospice, home, other and missing based on the recorded *address of death* in the ONS death certificate data. The ‘other’ category included deaths at respite care centres, nursing homes, deaths outside the home (eg, in a park or school). Deaths where the street address was not present were recorded as missing.

The year of death was assigned from the ONS death certificate date of death and the sex was assigned as the most commonly recorded sex from the HES data.

Age of death at last birthday was calculated by subtracting date of birth (HES record) from date of death (ONS record). Those who had died in the neonatal period (<28 days) were flagged on the death record. Seven age groups were created: neonate <28 days, 28 days–1 year, 1–5 years, 6–10 years, 11–15 years, 16–20 years and 21–25 years.

Self-reported ethnicity for each hospital episode was coded according to the 16 census groups;^15^ to prevent small numbers, these groups were collapsed into six ethnic groups as follows with the most commonly recorded ethnicity (from the six collapsed groups) assigned to each individual:

- White (white: British, white: Irish, other white)
- Black (black or black British: black Caribbean, black or black British: black African, black or black British: other black)
- Indian (Asian or Asian British: Indian)
- Pakistani (Asian or Asian British: Pakistani)
- Bangladeshi (Asian or Asian British: Bangladeshi)
- Other (Chinese, mixed or other)

The last known government office region of residence was assigned using the HES and ONS data for each individual.

An Index of Multiple Deprivation (IMD2010) Score^16^ was assigned to each individual based on last known lower super output area (LSOA) of residence. LSOAs are small geographical areas with a population from 1000 to 3000 individuals. Five population-weighted categories were created (category 1—least deprived) based on the IMD Scores with approximately 20% of the population living in each category.

The life-limiting condition diagnoses were grouped according to 11 diagnostic groups (neurology, haematology, oncology, metabolic, respiratory, circulatory, gastrointestinal, genitourinary, perinatal, congenital and other).^14^ The most common diagnostic group across all inpatient records for each individual was calculated with ties resolved by giving preference to later diagnoses.

### Analysis

To avoid missing data issue at the start of the study time period, children were included in the analyses 2003 onwards.

Temporal trends in place of death were plotted as a visual assessment of change over time. Place of death was described by sex, age group, ethnicity, government office region, deprivation category and main diagnostic group.

The association between the place of death and key clinical (diagnostic group) and demographic (age, sex, ethnic group, deprivation status and region) variables was assessed using multivariable multinomial logistic regression modelling comparing hospital death to both home and hospice deaths. Year of death was included in three equal epochs: 2003–2007, 2008–2012 and 2013–2017.^17^

All data manipulation was undertaken using Microsoft SQL server and statistical analysis using STATA V.15 (StataCorp, Collage Station, Texas, USA). Statistical significance was assumed at p≤0.05 (two-sided).

## Results

### Cohort

The total cohort of children with a life-limiting condition who had died from 2000 to 2017 was 53 518. After removal of those not resident in England (n=1512) and those who died before 2003 or after 2017 (n=7853) and those who died >25 years (n=4804), the final cohort for analyses was 39 349.

### Place of death

Overall 73% of deaths occurred in hospital, 6% in hospice and 16% at home. Five per cent died elsewhere or were missing place of death (table 1).

**Table 1:** Demographic characteristics of children and young people with a life-limiting condition who died*

	Hospital		Hospice		Home		Other or missing†		Total
	n	*%*	n	*%*	n	*%*	n	*%*	n
Total	28 753	*73*	2453	*6*	6269	*16*	1874	*5*	39 349
Sex									
Male	15 846	*73*	1332	*6*	3579	*16*	1066	*5*	21 823
Female	12 885	*74*	1121	*6*	2689	*15*	750	*4*	17 445
Age group at death									
Neonate (<28 days)	9511	*97*	163	*2*	171	*2*			9846
28 days to 1 year	5586	*71*	380	*5*	614	*8*	1301	*17*	7881
1–5 years	3498	*68*	488	*10*	1063	*21*	79	*2*	5128
6–10 years	1554	*57*	295	*11*	819	*30*	48	*2*	2716
11–15 years	1772	*59*	302	*10*	856	*29*	68	*2*	2998
16–20 years	3010	*63*	347	*7*	1266	*27*	138	*3*	4761
21–25 years	3822	*63*	478	*8*	1480	*25*	239	*4*	6019
Ethnic group									
White	18 017	*69*	1973	*8*	5030	*19*	949	*4*	25 969
Black	1937	*83*	73	*3*	187	*8*	136	*6*	2333
Pakistani	2521	*84*	99	*3*	301	*10*	93	*3*	3014
Indian	888	*81*	39	*4*	117	*11*	47	*4*	1091
Bangladeshi	544	*85*	16	*2*	61	*9*	22	*3*	643
Mixed/Chinese/other	2393	*78*	167	*5*	340	*11*	175	*6*	3075
Region									
North-East	1227	*71*	60	*4*	392	*23*	58	*3*	1737
North-West	4100	*75*	292	*5*	889	*16*	211	*4*	5492
Yorkshire and Humber	2745	*71*	361	*9*	663	*17*	122	*3*	3891
East Midlands	2212	*75*	153	*5*	512	*17*	87	*3*	2964
West Midlands	3270	*75*	291	*7*	661	*15*	164	*4*	4386
East of England	2435	*68*	295	*8*	710	*20*	120	*3*	3560
London	4874	*77*	267	*4*	775	*12*	423	*7*	6339
South-East	3631	*69*	457	*9*	985	*19*	219	*4*	5292
South-West	2136	*68*	250	*8*	626	*20*	119	*4*	3131
Deprivation category									
Category 1 (least deprived)	3179	*66*	431	*9*	1071	*22*	156	*3*	4837
Category 2	3866	*68*	454	*8*	1186	*21*	220	*4*	5726
Category 3	4851	*70*	495	*7*	1287	*19*	294	*4*	6927
Category 4	6096	*74*	507	*6*	1302	*16*	325	*4*	8230
Category 5 (most deprived)	7851	*78*	533	*5*	1339	*13*	409	*4*	10 132

*Those with missing deomgraphics are not presented due to small numbers.

†Combined to prevent small numbers.

**Figure 1:**
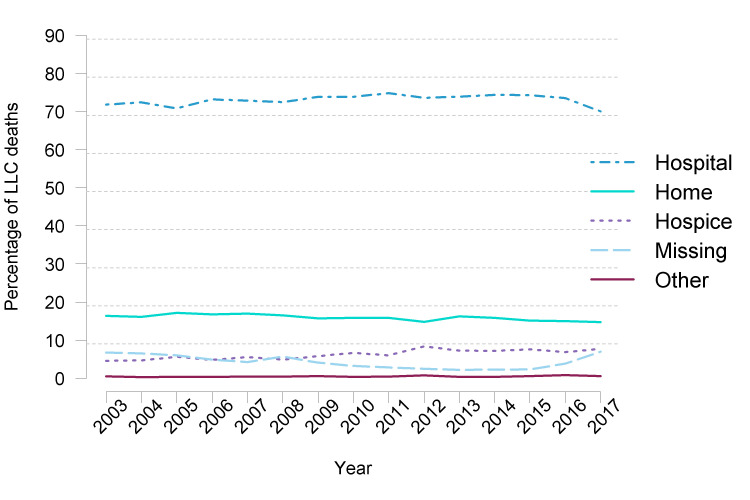
Trends in place of death of children and young people with a life-limiting condition in England from 2003 to 2017. LLC, life-limiting or life-threatening condition.

The percentage of deaths in hospital remained relatively static at just over 70% of deaths (figure 1). The percentage of children who died at home remained relatively static at around 15%–16%. This was in contrast to deaths in hospices which rose from 5% to 8% during the period of this study.

Place of death varied considerably by age with 97% of neonates and 71% of those aged 28 days to 1 year dying in hospital and only 2% and 8%, respectively, dying at home (table 1). The percentage of children dying at home peaks at 30% in those aged 6–10 years. Deaths in hospices were also most common, at 11%, in the 6–10 years age group.

A lower percentage of white children died in hospital (69%) when compared with the other ethnic groups where between 78% (Chinese/mixed/other) to 85% (Bangladeshi) died in hospital.

The highest percentage of deaths in hospital were in London (77%) with the lowest in the South-West (69%). Yorkshire and the Humber and the South-East had the highest percentage of hospice deaths (9%) and the North-East the lowest (4%). Conversely the highest percentage of home deaths was also in the North-East (23%) and the lowest in London (12%).

There is a linear trend with deprivation, the more deprived being more likely to die in hospital.

Table 2 shows the distribution of place of death by diagnostic category. The percentage of children with cancer who died in hospital was 44% with the next lowest metabolic at 61%. Home deaths were also highest among children who had cancer (41%) and were lowest among those who had perinatal conditions (2%). Children with cancer (14%), metabolic conditions (13%), other conditions (10%) or neurological conditions (9%) had the highest percentage of hospice deaths.

**Table 2:** Clinical characteristics of children and young people with a life-limiting condition who died

	Hospital			Hospice	Home		Other or missing*		Total
Cancer	3515	*44*	1075	*14*	3224	*41*	100	*1*	7914
Metabolic conditions	830	*61*	179	*13*	306	*23*	36	3	1351
Neurological conditions	3387	*68*	458	*9*	965	*19*	184	*4*	4994
Other conditions	530	*71*	73	*10*	92	*12*	51	*7*	746
Circulatory conditions	8276	*81*	425	*4*	917	*9*	621	*6*	10 239
Genitourinary conditions	1775	*82*	44	*2*	196	*9*	160	*7*	2175
Respiratory conditions	1766	*82*	69	3	243	*11*	86	*4*	2164
Haematological conditions	559	*85*	13	*2*	63	*10*	20	3	655
Congenital conditions	1074	*86*	20	*2*	105	*8*	46	*4*	1245
Gastrointestinal conditions	475	*88*	11	*2*	25	*5*	30	*6*	541
Perinatal conditions	6566	*90*	86	*1*	133	*2*	540	*7*	7325

*Combined due to small numbers.

The results of the multivariable multinomial logistic regression model comparing dying at home or in a hospice compared with dying in hospital are shown in table 3. The reference group for comparison throughout this section is hospital.

**Table 3:** Multivariable multinomial logistic models (n=34 425)

	Hospice versus hospital				Home versus hospital			
	RR	95% CI		P value	RR	95% CI		P value
Age at death								
Neonate	0.4	0.3	0.5		0.2	0.2	0.3	
28 days to 1 year	REF				REF			
1–5 years	1.1	0.9	1.3	<0.001	1.6	1.4	1.8	<0.001
6–10 years	1.1	0.9	1.3	<0.001	2.2	1.9	2.5	<0.001
11–15 years	1.0	0.8	1.2	0.002	2.0	1.7	2.3	<0.001
16–20 years	0.7	0.6	0.8	0.242	1.7	1.5	1.9	<0.001
21–25 years	0.8	0.6	0.9	0.956	1.6	1.4	1.8	<0.001
Sex								
Male	REF				REF			
Female	1.0	0.9	1.1	0.286	0.9	0.9	1.0	0.104
Ethnic group								
White	REF				REF			
Black	0.5	0.4	0.6	<0.001	0.5	0.4	0.6	<0.001
Pakistani	0.3	0.3	0.4	<0.001	0.5	0.5	0.6	<0.001
Indian	0.5	0.3	0.6	<0.001	0.6	0.5	0.7	<0.001
Bangladeshi	0.3	0.2	0.6	<0.001	0.6	0.4	0.8	<0.001
Mixed/Chinese/other	0.7	0.6	0.8	<0.001	0.6	0.5	0.7	<0.001
Missing	0.6	0.5	0.8	<0.001	0.7	0.6	0.9	<0.001
Deprivation category								
Category 1—least deprived	REF				REF			
Category 2	1.0	0.8	1.1	0.529	1.0	0.9	1.1	0.952
Category 3	0.9	0.8	1.0	0.145	0.9	0.8	1.0	0.147
Category 4	0.9	0.8	1.0	0.054	0.9	0.8	0.9	0.01
Category 5—most deprived	0.8	0.7	0.9	0.003	0.8	0.7	0.8	<0.001
Government office region								
London	REF				REF			
North-East	0.7	0.6	1.0	0.047	1.7	1.4	1.9	<0.001
North-West	1.2	01.0	1.4	0.062	1.2	1.1	1.4	0.002
Yorkshire and Humber	2.3	1.9	2.7	<0.001	1.4	1.2	1.6	<0.001
East Midlands	1.0	0.8	1.3	0.933	1.1	1.0	1.3	0.118
West Midlands	1.6	1.4	12.0	<0.001	1.2	1.1	1.4	0.003
East of England	1.8	1.5	2.1	<0.001	1.4	1.2	1.6	<0.001
South-East	1.8	1.5	2.2	<0.001	1.3	1.2	1.5	<0.001
South-West	1.7	1.4	2.1	<0.001	1.4	1.3	1.6	<0.001
Year of death								
2003–2007	REF				REF			
2008–2012	1.5	1.3	1.6	<0.001	1.1	1.04	1.2	0.003
2013–2017	1.7	1.5	1.9	<0.001	1.2	1.07	1.3	0.003
Main diagnostic group								
Congenital conditions	REF				REF			
Circulatory conditions	0.3	0.2	0.5	<0.001	0.6	0.5	0.7	<0.001
Gastrointestinal conditions	0.4	0.2	0.7	0.005	0.3	0.2	0.5	<0.001
Genitourinary conditions	0.4	0.3	0.6	<0.001	0.6	0.5	0.7	<0.001
Haematological conditions	0.4	0.2	0.7	0.003	0.6	0.5	0.8	0.004
Metabolic conditions	3.7	3.0	4.6	<0.001	2.1	1.8	2.5	<0.001
Neurological conditions	2.0	1.7	2.3	<0.001	1.3	1.2	1.5	<0.001
Cancer	4.4	43.8	5.1	<0.001	4.0	3.6	4.4	<0.001
Other conditions	2.4	1.8	3.1	<0.001	1.2	0.9	1.5	0.134
Perinatal conditions	0.3	0.3	0.4	<0.001	0.4	0.3	0.5	<0.001
Respiratory conditions	0.6	0.4	0.8	0.002	0.7	0.6	0.8	<0.001

NICE, National Institute for Health and Care Excellence; REF, reference group; RR, relative risk.

Hospice versus hospital: Neonates were less likely than those aged 28 days to <1 year to die in hospice and those aged 1–10 years were more likely to die in a hospice than those aged 28 days to 1 year. However, those aged 11–25 years were no more likely than the those aged 28 days to <1 year to die in a hospice.

Children from all the ethnic minority groups were significantly less likely to die in hospice with children of Bangladeshi origin being the least likely to die in a hospice (RR 0.3, 95% CI 0.2 to 0.6) compared with white children. Those in the most deprived group were also less likely to die in a hospice compared with the least deprived (RR 0.8, 95% CI 0.7 to 0.9). There were some geographical differences, with those who died in Yorkshire and Humber more likely than those in London to die in a hospice (RR 2.3, 95% CI 1.9 to 2.7). Those in the North-East were less likely than those in London to die in a hospice (RR 0.7, 95% CI 0.6 to 1.00).

Those who died after 2008 were more likely than those who died before 2008 to die in a hospice.

Children with a cancer (RR 4.4, 95% CI 3.8 to 5.1), neurological (RR 2.0, 95% CI 1.7 to 2.3) or metabolic (RR 3.7, 95% CI 3.0 to 4.6) diagnosis were more likely than those with a congenital diagnosis to die in a hospice.

There are some similarities in the home versus hospice component of the model for sex, ethnic group, deprivation and trends over time. The key differences in this comparison were that all age groups over 1 year were more likely than the 28 days to <1 year old group to die at home. Children from all other regions were more likely than those living in London to die at home compared (including the North-East, RR 1.7, 95% CI 1.4 to 1.9, in contrast to the results for hospice compared with hospital).

## Discussion

The majority of children with a life-limiting condition in England continue to die in a hospital setting. There is some evidence of an increase in hospice deaths since the government report ‘Better Care, Better Lives’ in 2008^18^ but there is still a relatively small number of deaths (<200) in hospices each year. The most recent national children’s hospice data collection showed that only 21% of their caseload who died, died in the hospice.^19^

A higher proportion died in hospital in the present study than in studies from other countries^20 21^ which may not be surprising given the different models of provision of palliative care and funding of healthcare systems across the world. The proportion of deaths at home are similar to figures from the USA (10.1% in 1989 rising to 18.2% in 2003)^22^ and Portugal (19.4%).^23^

Palliative care input has been associated with more children dying outside the hospital; a national study from England and Wales of 7709 children who died after being discharged from paediatric intensive care units showed that children who had palliative care recorded at the time of discharge were eight times more likely to die in the community than children who were not referred to palliative care.^11^ Likewise the study by Chang *et al*, showed that those who had palliative care were less likely to die in hospital.^20^ A study from Germany showed that of children who received specialist paediatric palliative care, 84% died at home with 96% in their preferred place.^24^ The general consensus among studies to date is that home is the preferred place of death, although not all families prefer home deaths; preferences vary over time and the research base consists of small studies which were prone to selection bias.^10^

Children with cancer were much more likely than other children to die at home or in a hospice. In England, children with cancer are treated and managed under a different model of care with palliative care being provided by specialists, including the paediatric oncology outreach nurses in most principal treatment centres.^25^ A recent national study showed that from 1993 to 2014, among children who died from cancer in England, those dying at home remained static at approximately 40%; hospital deaths decreased slightly from >50% to 45% and hospice deaths increased from 6% to 13%.^26^ An international study^21^ highlighted large variations in place of death between countries and that children with conditions other than cancer were less likely to die at home.

Age has been shown in several studies to be associated with dying at home with infants less likely than older children to die outside hospital.^22 27^ The predominance of hospital death in the neonatal group may highlight the unpredictability of their prognosis and the additional challenges of offering choice of place of death in neonatal care.^28^

This study highlighted the differences in place of death for children from a minority ethnic group. This has been shown in other studies.^20 26^ There is little evidence on preferences of place of care in these populations and differences could possibly be due to access to healthcare services, divergent cultural attitudes or differing levels of financial or social support within a patient’s family or social network.^22^ Importantly, services must be flexible enough to meet the needs of all children and their families.^17 29^

### Strengths and limitations

This study used a whole population data set to identify children with a life-limiting condition and national death registration records. There can be delays in the registration of deaths for children if the coroner is involved so the recent years of data may be incomplete.

It was not possible to identify those children who had and had not received palliative care prior to death. There were no data on preferred place of death available for this cohort of children.

## Conclusions

Despite small increases in hospice deaths over the last 15 years the vast majority of children with a life-limiting condition die in the hospital setting. Further research is needed on preferences for place of death especially in children with conditions other than cancer. Paediatric palliative care services should be funded adequately to enable equal access across all settings, diagnostic groups and geographical regions.

## Data Availability

Data may be obtained from a third party and are not publicly available. Data are available from NHS Digital.
