# Supplementary material for: Place of death of children and young adults with a life-limiting condition in England: a retrospective cohort study
Source: Arch Dis Child. 2020 Dec 21;106(8):780–5. doi: 10.1136/archdischild-2020-319700 (PMC8311108; doi:10.1136/archdischild-2020-319700)
Supplement: Supplementary data [file archdischild-2020-319700supp001.pdf]

Supplementary Table 1

: ICD-10 diagnostic coding framework used to identify and categorise children with life-limiting conditions.

Diagnostic Group	ICD-10 Numbers
**Neurology**	A17 A810 A811 F803 F842 G10 G111 G113 G12 G20 G230 G238 G318 G319 G35 G404 G405 G600 G601 G702 G709 G710 G711 G712 G713 G800 G808 G823 G824 G825 G934 G936 G937
**Haematology**	B20 B21 B22 B23 B24 D561 D610 D619 D70 D761 D81 D821 D83 D891
**Oncology**	C D444 D48 (Central Nervous System: C70,C71,C72, D33, D43)
**Metabolic**	E310 E348 E702 E71 E72 E74 E75 E76 E77 E791 E830 E880 E881
**Respiratory**	E84 J841 J96 J984
**Circulatory**	I21 I270 I42 I613 I81
**Gastrointestinal**	K550 K559 K72 K74 K765 K868
**Genitourinary**	N17 N18 N19 N258 (Early stage (1-3) renal:N181, N182, N183)
**Perinatal**	P101 P112 P210 P285 P290 P293 P350 P351 P358 P371 P524 P525 P529 P832 P912 P916 P960
**Congenital**	Q000 Q01 Q031 Q039 Q040 Q042 Q043 Q044 Q046 Q049 Q070 Q200 Q203 Q204 Q206 Q208 Q213 Q232 Q218 Q220 Q221 Q224 Q225 Q226 Q230 Q234 Q239 Q254 Q256 Q262 Q264 Q268 Q282 Q321 Q336 Q396 Q410 Q419 Q437 Q442 Q445 Q447 Q601 Q606 Q614 Q619 Q642 Q743 Q748 Q750 Q772 Q773 Q774 Q780 Q785 Q792 Q793 Q804 Q81 Q821 Q824 Q858 Q860 Q870 Q871 Q872 Q878 Q91 Q920 Q921 Q924 Q927 Q928 Q932 Q933 Q934 Q935 Q938 Q952
**Other**	H111 H498 H355 M313 M321 M895 T860 T862 Z515
